# Exploring the role of artificial intelligence, large language models: Comparing patient‐focused information and clinical decision support capabilities to the gynecologic oncology guidelines

**DOI:** 10.1002/ijgo.15869

**Published:** 2024-08-20

**Authors:** Lee Reicher, Guy Lutsker, Nadav Michaan, Dan Grisaru, Ido Laskov

**Affiliations:** ^1^ Department of Gynecologic Oncology Lis Hospital for Women, Tel Aviv Medical Center Tel Aviv Israel; ^2^ Sackler School of Medicine, Department of Gynecology Tel Aviv University Tel Aviv Israel; ^3^ Department of Molecular Cell Biology Weizmann Institute of Science Rehovot Israel; ^4^ Department of Computer Science and Applied Mathematics Weizmann Institute of Science Rehovot Israel

**Keywords:** artificial intelligence, ChatGPT, gynecologic cancer management, health communication, large language model, patient education

## Abstract

Gynecologic cancer requires personalized care to improve outcomes. Large language models (LLMs) hold the potential to provide intelligent question‐answering with reliable information about medical queries in clear and plain English, which can be understood by both healthcare providers and patients. We aimed to evaluate two freely available LLMs (ChatGPT and Google's Bard) in answering questions regarding the management of gynecologic cancer. The LLMs' performances were evaluated by developing a set questions that addressed common gynecologic oncologic findings from a patient's perspective and more complex questions to elicit recommendations from a clinician's perspective. Each question was presented to the LLM interface, and the responses generated by the artificial intelligence (AI) model were recorded. The responses were assessed based on the adherence to the National Comprehensive Cancer Network and European Society of Gynecological Oncology guidelines. This evaluation aimed to determine the accuracy and appropriateness of the information provided by LLMs. We showed that the models provided largely appropriate responses to questions regarding common cervical cancer screening tests and *BRCA*‐related questions. Less useful answers were received to complex and controversial gynecologic oncology cases, as assessed by reviewing the common guidelines. ChatGPT and Bard lacked knowledge of regional guideline variations, However, it provided practical and multifaceted advice to patients and caregivers regarding the next steps of management and follow up. We conclude that LLMs may have a role as an adjunct informational tool to improve outcomes.

## Synopsis

ChatGPT and Bard provide appropriate responses to patient's perspective gynecologic oncologic questions, but is less useful for complex questions compared with the National Comprehensive Cancer Network/European Society of Gynecological Oncology guidelines.

## 1 INTRODUCTION

Natural Language Processing (NLP) falls under the realm of artificial intelligence (AI) and focuses on the interplay between human language and computers. Recent years have witnessed substantial advancements in NLP, largely attributed to the emergence of intricate deep‐learning models. These models have significantly enhanced the execution of NLP undertakings, such as processing text and speech, leading to remarkable demonstrations of their capabilities. Two noteworthy illustrations of these achievements are ChatGPT, which emerged in November 2022 when OpenAI introduced this publicly accessible online chatbot, and Google's Bard (Google, Menlo Park, CA, USA), which provides free conversational AI services that use a set of deep‐learning algorithms to respond to questionnaires.

Serving as a straightforward “input–output” interface, these large language models (LLMs) ingested a staggering amount of textual data—approximately 57 billion words and 175 billion parameters—sourced from various outlets including the internet and books. This extensive dataset empowers LLM to adeptly handle a wide spectrum of prompts spanning diverse domains, including the medical field. Its proficiency encompasses language‐related tasks as diverse as crafting articles^1^ to answering clinical questions.^2^

LLMs have proven useful in various medical applications. They create accurate lists of potential diagnoses, support clinical decision making, improve clinical decision support, and provide valuable insights for cancer screening. Furthermore, LLMs have been harnessed for astute question‐answering endeavors, furnishing dependable information regarding diseases and medical inquiries. Their proficiency in delivering accurate responses to clinical questions in plain and understandable English stands out, catering to both healthcare practitioners and patients.^3^

In the realm of medical documentation, LLMs have demonstrated their effectiveness by generating patient clinical letters, radiology reports, medical notes, and discharge summaries. This has led to enhanced efficiency and precision for healthcare providers, streamlining their tasks.^4^

While observing its capability, it is important to characterize its limitations. An LLM is only as good as its derived training data. These data are potentially biased, unreliable, and are not necessarily up to date. According to OpenAI, the model has “limited knowledge of the world and events after 2021.”

Users must take its responses with a degree of faith because it does not list sources for its information. A seemingly satisfactory LLM risks misleading without proper warning.

To obtain medical advice, individuals may explore informational sources, including those on the internet, or communicate with clinicians.

In the near future, LLMs will probably become the new standard for healthcare professionals and patients to access medical information. These new tools are being evaluated in various fields in medicine,^2^, ^3^, ^4^, ^5^, ^6^, ^7^, ^8^, ^9^ but there is no information available about their quality in gynecologic oncology‐related topics.

Our aim was to assess the responses provided by two freely available LLMs (ChatGPT and Bard) to a range of questions related to gynecologic oncology.

## 2 MATERIALS AND METHODS

We evaluated the two models' performance both as a patient‐facing information platform for answering common questions and as a tool to guide clinicians in controversial or complex cases within the field of gynecologic oncology.

To conduct the study, we developed a set of three questions that addressed common gynecologic oncology findings from a patient's perspective. Additionally, we included three questions that focused on more complex cases to elicit recommendations from a clinician's perspective. Each question was presented to the LLM's interface, and the responses generated by the AI model were recorded.

To evaluate the responses, we assessed their adherence to the National Comprehensive Cancer Network (NCCN) and European Society of Gynecological Oncology (ESGO) guidelines. This evaluation aimed to determine the accuracy and appropriateness of the information provided by the LLMs.

By comparing the AI‐generated responses with common guidelines, we aimed to assess the potential utility of LLMs as a patient information platform and as a resource for guiding clinicians in challenging or controversial cases within the realm of gynecologic oncology. The results of this study will provide valuable insights into the capabilities and limitations of AI language models in this specialized medical field.

The questions are highlighted as subtitles, the LLMs' responses are entered in quotations. In accordance with the journal's guidelines, we will provide our data for independent analysis by a selected team by the Editorial Team for the purposes of additional data analysis or for the reproducibility of this study in other centers if such is requested.

## 3 RESULTS

### 3.1 From the clinician's perspective—controversial cases within the field of gynecologic oncology

**You and I are gynecologist‐oncologists discussing a 50‐year‐old patient with surgically staged International Federation of Gynecology & Obstetrics (FIGO) Stage 1a, Grade 3 endometrioid adenocarcinoma. What are your recommendations regarding adjuvant therapy?**

ChatGPT

1. Adjuvant radiation therapy: Adjuvant radiation therapy may be considered if there are certain high‐risk features present, such as deep myometrial invasion or involvement of the cervix. Radiation therapy can be administered externally (external beam radiation, EBRT) or internally (brachytherapy) to target the tumor bed and reduce the risk of local recurrence. EBRT involves the delivery of high‐energy X‐rays to the pelvis to destroy any remaining cancer cells and reduce the risk of local recurrence. It may be recommended if there are certain high‐risk features such as lymphovascular space invasion (LVSI), deep myometrial invasion, or involvement of the cervix. Brachytherapy, also known as internal radiation therapy, involves the placement of radioactive sources directly into the vagina or uterus to deliver a targeted dose of radiation to the tumor bed. It is often used as an adjuvant treatment in early‐stage endometrial cancer, particularly when there are high‐risk features. Brachytherapy can help to reduce the risk of recurrence in the vagina or uterus.
2. Adjuvant chemotherapy: Systemic chemotherapy may be recommended if there are factors indicating a higher risk of recurrence. These factors may include LVSI, positive surgical margins, or other adverse features.
3. Hormonal therapy: In cases where the endometrial cancer cells are hormone‐receptor‐positive, hormonal therapy may be considered. This treatment involves using medications such as tamoxifen or aromatase inhibitors to block the effects of estrogen on cancer cells and reduce the risk of recurrence.

Bard

This scenario presents a complex decision making process, as Stage 1a endometrioid adenocarcinoma typically carries a favorable prognosis, even with high‐grade features like Grade 3. However, Grade 3 does raise some concerns and warrants careful consideration of adjuvant therapy options. Here is how we can approach this discussion:

1. Consider additional risk factors:
  - Lymphovascular space invasion: Presence or absence of LVSI is crucial. Positive LVSI significantly increases the risk of recurrence and justifies adjuvant therapy.
  - Molecular profiling: The recently introduced 2023 FIGO staging incorporates molecular classification. Testing for mismatch repair deficiency and POLE (polymerase ε) mutations can identify tumors with a very low risk of recurrence, potentially making adjuvant therapy unnecessary.
  - Other factors: Patient age, overall health, preferences, and fertility desires should also be taken into account.
2. Weigh the benefits and risks of adjuvant therapy:
  - Benefits: Reduce the risk of recurrence and improve overall survival, especially if additional risk factors are present.
  - Risks: Potential side effects of treatment, such as fatigue, nausea, and long‐term complications, depending on the specific therapy chosen.
3. Treatment options:
  - Observation: For patients with truly low‐risk tumors (negative LVSI, mismatch repair proficient, POLE wild‐type), observation with close surveillance may be appropriate.
  - Vaginal brachytherapy: Offers excellent local control with minimal side effects and could be considered for some patients with positive LVSI or other intermediate‐risk features.
  - Pelvic radiotherapy: Traditionally used for adjuvant therapy, but may be associated with more side effects compared with brachytherapy.
  - Hormonal therapy: Progestin therapy may be considered for high‐risk features, but its role in the adjuvant setting is still evolving.
4. Shared decision making:
  - Ultimately, the decision regarding adjuvant therapy should be made jointly by you, the patient, and potentially other members of the multidisciplinary team, considering all the individual factors and weighing the risks and benefits of each option.
5. Additional points to consider:
  - The recent 2023 FIGO staging system incorporates molecular classification, which can further refine risk assessment and potentially guide adjuvant therapy decisions.
  - Ongoing research is evaluating the role of newer therapies, such as immunotherapy, in the adjuvant setting for endometrial cancer.

Guidelines:

NCCN—Brachytherapy preferred or observation if no myoinvasion or consider EBRT if either age ≥70 years or LVSI.^10^

ESGO—If molecular classification unknown and LVSI negative or focal brachytherapy or observation. If LVSI, a substantial adjuvant EBRT, is recommended, additional chemotherapy can be considered.^11^

Comments:

In the case of contacting an expert physician, certain explanations are unnecessary and time consuming, such as explaining the meaning of adjuvant radiation therapy.

Compared with the NCCN and ESGO guidelines, age is not mentioned by ChatGPT. Molecular classification, which is important for prognosis assessment and treatment adjuvant decisions, is only mentioned in the ESGO guidelines and by Bard. Optional adjuvant chemotherapy is mentioned in both ChatGPT and ESGO guidelines but not at the NCCN guidelines and in Bard.

**You and I are gynecologist‐oncologists discussing a patient with cervical cancer Stage IA1 no LVSI with negative margins on cone biopsy. What are your recommendations?**

ChatGPT

1. Pathologic findings: The absence of LVSI and negative margins on the cone biopsy suggest a lower risk of disease spread beyond the cervix. This is a positive prognostic factor.
2. Multidisciplinary approach: Treatment decisions for cervical cancer often involve a multidisciplinary team, including gynecologic oncologists, radiation oncologists, and medical oncologists. It is important to consider the patient's overall health, desire for fertility preservation, and other individual factors when determining the most appropriate treatment plan.
3. Surgery versus conservative management: In Stage IA1 cervical cancer, several treatment options can be considered. One approach is to proceed with a definitive surgery, such as a radical hysterectomy or trachelectomy, depending on the patient's desire for fertility preservation. Alternatively, if fertility preservation is a priority and the patient wishes to conceive in the future, close surveillance with regular follow up and potential fertility‐sparing procedures (such as cervical conization or simple trachelectomy) may be considered.
4. Adjuvant therapy: In the absence of adverse features such as positive margins or LVSI, adjuvant therapy may not be routinely indicated. However, it is crucial to thoroughly evaluate the patient's case and consider any additional risk factors or characteristics that may warrant adjuvant treatment, such as deep stromal invasion or high‐grade histology. Discussion within a multidisciplinary tumor board can help determine if any additional treatment is necessary.
5. Follow up and surveillance: Regardless of the treatment approach chosen, regular follow up with close surveillance is essential. This includes periodic pelvic examinations, cervical smear tests, and imaging studies as recommended by clinical guidelines. Ensuring ongoing compliance with surveillance measures is crucial for early detection of potential recurrence or development of new lesions.

Bard

1. Consider patient preferences and future fertility desires:
  - Discuss the patient's priorities and goals regarding fertility preservation and future childbearing plans.
2. Treatment options:
  - Observation: For patients with truly low‐risk features like this case, close observation with regular cervical smear tests and colposcopy can be a viable option. Studies have shown excellent long‐term survival rates with this approach.
  - Radical trachelectomy: This minimally invasive surgery removes the cervix and upper part of the vagina while preserving the uterus and ovaries. It offers an option for women desiring future pregnancy.
  - Radical hysterectomy: This surgery removes the cervix, uterus, upper vagina, and surrounding lymph nodes. It is a definitive treatment with high cure rates but precludes future pregnancy.
  - Laparoscopic pelvic and para‐aortic lymphadenectomy: This minimally invasive surgery removes lymph nodes to assess for potential spread of cancer. Although not curative in itself, it can provide valuable prognostic information and guide further management.
3. Factors influencing treatment choice:
  - Depth of invasion: Although Stage IA1, the exact depth of invasion within the 3‐mm limit might influence some decisions.
  - Cone biopsy technique: Was it loop electrosurgical excision procedure (LEEP), cold knife conization, or another method? This can affect the confidence in margin assessment.
  - Patient age and overall health: Younger age and good health might favor more aggressive surgery, while older age or comorbidities might favor observation or less invasive approaches.
4. Shared decision making:
  - Ultimately, the best treatment option should be chosen collaboratively with the patient, considering their individual circumstances, preferences, and risk tolerance. It is crucial to discuss the benefits and risks of each option, including potential side effects and impact on future fertility.
5. Additional points to consider:
  - In some cases, adjuvant therapy (pelvic radiation or brachytherapy) might be recommended if specific concerns arise, such as positive lymph nodes identified on lymphadenectomy.
  - Ongoing research is evaluating the role of novel therapies, such as immunotherapy, in early‐stage cervical cancer.

Guidelines:

NCCN—Observe if inoperable or extrafascial hysterectomy if operable.^12^

ESGO—Cone is considered a definitive treatment. No LN staging needed.^13^

Comments:

In this conversation with an expert, the answer remains at a high level. Both the ChatGPT and Bard elaborate on various issues including LVSI as a prognostic factor, fertility preservation desire with treatment options, and surveillance. Both models suggest radical hysterectomy, which is considered as overtreatment for Stage 1A1, LVSI‐negative cervical cancer.

**You and I are gynecologist‐oncologists discussing a 60‐year‐old patient with low‐grade serous ovarian carcinoma Stage II. After cytoreductive surgery, what are your recommendations for adjuvant therapy?**

ChatGPT

1. Platinum‐based chemotherapy: The most common adjuvant treatment for low‐grade serous ovarian carcinoma is platinum‐based chemotherapy. This typically involves a combination of carboplatin and paclitaxel. This regimen has shown effectiveness in reducing the risk of disease recurrence and improving overall survival.
2. Duration of chemotherapy: The duration of chemotherapy can vary. Generally, adjuvant chemotherapy is recommended for a total of six cycles, given every 3 weeks. However, individual patient factors and tolerability should be taken into account when determining the exact duration of treatment.
3. Hormonal therapy: Low‐grade serous ovarian carcinoma has been found to be associated with mutations in the KRAS/BRAF pathway. As a result, targeted therapies aimed at inhibiting this pathway, such as hormonal therapies like letrozole or tamoxifen, may be considered in certain cases. However, the efficacy of hormonal therapy in this specific context is still being studied, and further research is needed.
4. Bevacizumab: Bevacizumab is an anti‐angiogenic agent that can be considered as an option for adjuvant therapy in select cases. It may be particularly beneficial if residual disease is present after cytoreductive surgery or in cases with high‐risk features. However, its use should be discussed and carefully evaluated with consideration for potential side effects and individual patient factors.

Bard

1. Extent of cytoreduction:
  - Optimal (R0): No visible macroscopic residual disease. This scenario favors observation in many cases.
  - Suboptimal (R1): Microscopic residual disease. Adjuvant therapy is generally recommended.
  - Macroscopic residual disease (R2): Visible residual disease remaining after surgery. More intensive therapy, potentially including additional surgery, is often considered.
2. Additional risk factors:
  - Lymphovascular space invasion: Positive LVSI increases the risk of recurrence and justifies adjuvant therapy.
  - Diaphragmatic involvement: Involvement of the diaphragm further elevates the risk and strengthens the case for adjuvant therapy.
  - Patient age and overall health: Younger age and good health favor more aggressive treatment approaches.
3. Treatment options:
  - Observation: For patients with optimal cytoreduction (R0) and no other high‐risk features, observation with close surveillance can be considered.
  - Platinum‐based chemotherapy: Traditionally the mainstay of adjuvant therapy for low‐grade serous ovarian cancer, particularly with suboptimal cytoreduction (R1 or R2). Regimens like carboplatin and paclitaxel are commonly used.
  - PARP (poly [ADP‐ribose] polymerase) inhibitors: These targeted agents like olaparib have shown promising results in clinical trials for adjuvant therapy in patients with *BRCA* mutations.
  - Hormonal therapy: Progestin therapy may be considered in some cases, but its role in the adjuvant setting is still evolving.
4. Individualized approach:
  - The optimal treatment plan should be tailored to the patient's specific situation, considering all the aforementioned factors.
5. Additional points to consider:
  - Ongoing research is evaluating newer treatment options and refining patient selection for different therapies.
  - Shared decision making with the patient is crucial, discussing the benefits and risks of each option and aligning with their preferences and goals.

Guidelines:

NCCN—Systemic therapy with chemotherapy or hormonal therapy followed by maintenance letrozole or other hormonal therapy.^14^

ESGO—3‐weekly carboplatin AUC5/6 plus paclitaxel 175 mg/m^2^, six cycles.^15^

Comments:

The guidelines, ChatGPT and Bard recommendations agree on the use of platinum‐based chemotherapy. Hormonal treatment is suggested by both of the models and the NCCN guidelines but not by the ESGO guidelines. Bard's response included a more in‐depth discussion of the extent of cytoreduction, whereas ChatGPT did not mention it. ChatGPT mentioned bevacizumab as a potential treatment option based on its evaluation in clinical trials for ovarian cancer, including low‐grade serous carcinoma. However, neither NCCN nor ESGO guidelines mentioned bevacizumab in the adjuvant setting for low‐grade serous carcinoma.

### 3.2 Patient‐facing platform for addressing common gynecologic oncology concerns

**What are the recommendations for screening tests routine for cervical cancer?**

ChatGPT

1. Pap smear (Pap Test): The cervical smear test is the most common screening test for cervical cancer. It involves collecting cells from the cervix and examining them under a microscope to detect any abnormal changes. Starting at the age of 21 years, most guidelines recommend having a cervical smear every 3 years for women with a cervix.
2. HPV testing: HPV is a common sexually transmitted infection that can lead to cervical cancer. HPV testing is often done in conjunction with a cervical smear for women aged 30 years and older. It detects the presence of high‐risk HPV types that may cause cervical cell changes. If both the cervical smear and HPV test results are normal, screening intervals can be extended to every 5 years.
3. Co‐testing: In some cases, both cervical smear and HPV testing (co‐testing) may be performed together for women aged 30–65 years. If both tests are negative, the screening interval can be extended.
4. HPV vaccination: Vaccination against certain high‐risk HPV types is recommended for both females and males between the ages of 9 and 26 years. The HPV vaccine is most effective when administered before the onset of sexual activity.
5. Screening cessation: In general, cervical cancer screening can be stopped at the age of 65–70 years if a woman has had regular screenings in the past and her recent results are consistently normal. However, individual circumstances may vary, so it is important to consult with a healthcare provider to determine the appropriate screening schedule.

Bard

1. HPV test:
  - Recommended age: Starting at age 25 (some organizations recommend 30).
  - Frequency: Every 5 years (if negative).
  - What it detects: Presence of high‐risk HPV types linked to cervical cancer.
  - Benefits: Highly sensitive and can detect precancerous changes before symptoms appear.
2. Pap test (Papanicolaou smear [cervical smear]):
  - Recommended age: Starting at age 21 years (some organizations recommend 30).
  - Frequency: Every 3 years (if negative), co‐testing with HPV every 5 years after age 30 years (some organizations recommend co‐testing starting at age 25).
  - What it detects: Abnormal cervical cells that could potentially become cancerous.
  - Benefits: Established and reliable test for detecting precancerous changes.
3. Additional considerations:
  - Women with a history of abnormal cervical smear tests or HPV infection may require more frequent screening.
  - Women who have received the HPV vaccine should still follow recommended screening guidelines.
  - Consult your healthcare provider for personalized screening recommendations based on your individual history and risk factors.
4. Here are some resources with detailed information on cervical cancer screening:
  - American Cancer Society: https://www.cancer.org/cancer/types/cervical‐cancer/detection‐diagnosis‐staging/cervical‐cancer‐screening‐guidelines.html
  - Centers for Disease Control and Prevention: https://www.cdc.gov/cancer/cervical/index.htm
  - National Cancer Institute: https://www.cancer.gov/types/cervical/hp

Comment:

Both of the models provide a simple explanation of the screening tests, discuss all the important points, such as co‐testing and the difference between HPV typing and cervical smear test, emphasize the importance of vaccination and indicate when screening tests should be stopped. For patients who wish to continue reading on their own, Bard provides additional resources.

**You are a gynecologist‐oncologist, I'm a healthy 30‐year‐old woman and have a positive Pap (low‐grade squamous intraepithelial lesions [LSIL]) and positive** **HPV** **16. What should I do next?**

ChatGPT

1. Colposcopy: Given the positive cervical smear and HPV 16 result, a colposcopy is strongly recommended. During this procedure, a colposcope (a specialized microscope) is used to closely examine the cervix for any abnormal areas.
2. Biopsy: If any abnormal areas are identified during the colposcopy, the gynecologist may perform a biopsy. This involves taking small samples of tissue from the cervix for further examination under a microscope.
3. Histologic evaluation: The biopsy samples will be sent to a pathology laboratory for histologic evaluation. A pathologist will analyze the tissue samples to determine the severity of the cellular changes and provide a definitive diagnosis.
4. Management based on histology and HPV 16: (a) LSIL/Cervical Intraepithelial Neoplasia Grade 1 (CIN1): If the biopsy confirms LSIL or CIN1, close observation with regular follow up is often recommended. This may involve repeat cervical smears at regular intervals (typically every 6–12 months) to monitor any changes. As you have tested positive for high‐risk HPV type 16, more frequent follow up and monitoring may be advised. (b) HPV‐related High‐Grade Lesions: If the biopsy reveals higher‐grade changes such as high‐grade squamous intraepithelial lesion or CIN Grade 2 or 3 (CIN2/3), further evaluation and treatment may be required. Options may include LEEP, cold knife conization, or other excisional procedures to remove the abnormal tissue.

Bard

A positive cervical smear (LSIL) and positive HPV 16 indicate LSIL, which are abnormal cell changes in the cervix. While HPV 16 is considered high risk and associated with cervical cancer, LSIL usually resolves on its own without developing into cancer.

1. However, your doctor will likely recommend further investigation to assess the situation and determine the appropriate management plan. This may involve:
  - Colposcopy: A magnified examination of the cervix using a special instrument.
  - Cervical biopsy: Taking a small tissue sample from the cervix for analysis.
2. Based on the results of these tests, your doctor will recommend the most appropriate management approach, which might include:
  - Observation: In many cases with LSIL, observation with regular cervical smear tests and colposcopy may be sufficient.
  - Loop electrosurgical excision procedure: A minimally invasive procedure to remove abnormal cervical tissue.
  - Other treatment options: Depending on the specific findings, other treatments like cryotherapy or laser ablation might be considered.
3. Remember:
  - Do not panic. LSIL is a common finding and often resolves on its own.
  - Schedule an appointment with your gynecologist or oncologist as soon as possible. Discuss your specific situation and receive personalized advice and guidance.
  - Follow your doctor's recommendations for further tests and management.
  - Maintain regular cervical cancer screening as advised by your doctor.
4. Here are some reliable resources you can refer to for additional information:
  - American Cancer Society: https://www.cancer.org/cancer/types/cervical‐cancer/detection‐diagnosis‐staging/cervical‐cancer‐screening‐guidelines.html
  - Centers for Disease Control and Prevention: https://www.cdc.gov/cancer/cervical/basic_info/screening.htm
  - National Cancer Institute: https://www.cancer.gov/types/cervical/hp

Comments:

Both of the models clearly guide the user to the appropriate next step, colposcopy, and even elaborate on what the women will experience and what her next steps will be. The answer was detailed and user‐friendly. This can help patients with similar conditions have less uncertainty. As with the previous task, Bard also provides links for further reading.

**You are a gynecologist‐oncologist, I'm a healthy 31‐year‐old woman and have recently discovered that I am a BRCA carrier. What are your recommendations?**

ChatGPT

1. Breast cancer screening:
  - Mammogram: Starting at a younger age than the general population (usually around age 25–30 years), you should begin regular screening mammograms. The frequency may vary based on your specific risk factors, but it is often recommended to have them every 6–12 months.
  - Breast magnetic resonance imaging (MRI): In addition to mammograms, breast MRI may be advised, typically starting at a similar age. This imaging method is more sensitive for detecting breast cancer in *BRCA* carriers.
  - Clinical Breast Examination: Regular clinical breast examinations by a healthcare provider should also be performed every 6–12 months.
2. Ovarian cancer screening:
  - Transvaginal ultrasound (TVUS): There is no perfect screening test for ovarian cancer in *BRCA* carriers. TVUS is one option, and it may begin around the age of 30–35 years. TVUS is used to look for any abnormal changes in the ovaries.
  - CA125 blood test: The CA125 blood test, which measures a protein often elevated in ovarian cancer, may be used in combination with TVUS. However, it is important to note that CA125 levels can be influenced by various factors and may not be specific enough for ovarian cancer screening.
3. Risk‐reducing surgery:
  - Prophylactic bilateral mastectomy: Consider discussing with your healthcare provider and a genetic counselor about the possibility of a prophylactic bilateral mastectomy (removal of both breasts) to reduce the risk of breast cancer. This decision depends on your personal circumstances and preferences.
  - Prophylactic oophorectomy: Removing the ovaries and fallopian tubes is recommended for *BRCA* carriers, usually after childbearing is completed or around the age of 35–40 years. This procedure significantly reduces the risk of ovarian cancer and may also reduce the risk of breast cancer in premenopausal individuals.

Bard

1. Seek professional guidance:
  - Schedule an appointment with a genetic counselor: They can explain the implications of your *BRCA* mutation, discuss your cancer risks, and guide you through appropriate screening and prevention options.
  - Consult a gynecologist‐oncologist: This specialist can tailor a personalized risk assessment and discuss specific recommendations for cancer screening and prevention strategies relevant to your situation.
2. Understand your increased cancer risks:
  - *BRCA* mutations increase the risk of certain cancers, including breast and ovarian cancer. However, it is important to remember that having a mutation does not guarantee that you will develop cancer.
  - Your genetic counselor and doctor can provide specific information about your individual risk based on your specific mutation and family history.
3. Cancer screening and prevention options:
  - Early and frequent screening: Depending on your risk assessment, your doctor may recommend starting mammograms and breast MRIs at a younger age and more frequently than usual. Regular pelvic examinations and transvaginal ultrasounds might also be advised.
  - Preventive surgery: Some women with *BRCA* mutations opt for risk‐reducing surgeries like prophylactic mastectomy or oophorectomy to significantly reduce their cancer risk. However, this is a major decision that requires careful consideration and discussion with your doctor.
  - Chemoprevention: For women with a high risk of breast cancer, medications like tamoxifen or raloxifene might be considered to further reduce the risk.
4. Additional resources and support:
  - National Cancer Institute: https://www.cancer.gov/about‐cancer/causes‐prevention/genetics/brca‐fact‐sheet
  - Susan G. Komen: https://www.komen.org/
  - Facing Our Risk of Cancer Hereditary Cancer Empowerment (FORCE): https://www.facingourrisk.org/
  - Bright Pink: https://www.brightpink.org/

Comment:

Overall, the answers were very informative and aligned with the guidelines. However, birth control pills were not mentioned as a known risk reduction option for ovarian cancer. Furthermore, Bard mentioned chemoprevention, an uncommon practice. Once again, the model refers to consulting with healthcare providers.

## 4 DISCUSSION

This exploratory study found that popular online AI models gave mostly appropriate answers to questions about common screening tests (cervical smear smear and HPV typing). However, the models provided less useful responses for complex and controversial gynecologic oncology cases, as assessed by the NCCN and ESGO guidelines.

Despite LLMs' growing popularity and performance, there is still a lack of studies evaluating their use in clinical practice, particularly in the gynecologic oncology field. A recent study evaluated the accuracy of ChatGPT in answering commonly asked questions pertaining to cervical cancer. The answers were scored by two gynecologic oncologists. ChatGPT accurately answered questions about cervical cancer prevention (91.7%), survivorship, and quality of life (93.8%), but performed less accurately for cancer diagnosis (71.4%) and treatment (33.3%).^16^

Previous studies assessed the accuracy of responses provided by virtual assistants and generative AI chatbots to voice queries related to gynecologic oncology. The correct answers for these questions were sourced from the UpToDate medical resource rather than from official guidelines.^17^, ^18^ In our study, we discuss the application of clinical decision support and patient medical queries using the most popular LLMs (ChatGPT and Bard) in comparison with the available NCCN and ESGO guidelines. ChatGPT and Bard both provided detailed and coherent recommendations across various scenarios, demonstrating comparable medical knowledge. In particular, both models exhibited a similar understanding of NCCN and ESGO guidelines. Although the models were aligned with guidelines in some instances, subtle discrepancies and the significance of expert consultation also emerged through the evaluation.

LLMs may be valuable to users who need preliminary information about routine screening tests. In general, their replies were nuanced, eloquent, well‐informed, and virtually error‐free. They may, therefore, have a promising role to play in patient education. Findings suggest the potential of interactive AI to assist clinical workflows by augmenting patient education and patient‐clinician communication around gynecologic oncology queries. An application of this type could offer simple answers to patients' questions related to routine screenings.

There are several limitations to this study. Gynecologic oncology is a wide field that is not covered by the preliminary list of simple questions in this study. AI accuracy and reliability are susceptible to training data limitations and prompt engineering. This study used the versions of ChatGPT and Bard available at the time of this analysis and did not assess other AI language models. Finally, the AI tools' responses did not include references to evidence to support any statements. The AI answers were compared with the NCCN and ESGO guidelines and were not validated by answers provided by professional gynecologic oncologists.

In conclusion, ChatGPT and Bard have demonstrated promising prospects in clinical practice for both clinicians and patients. For clinicians, these models provide useful insights into complex gynecologic oncology cases, although expert validation remains essential. From the patient perspective, the models offer accurate and comprehensible responses to routine screening questions and general gynecologic oncology concerns, potentially aiding in better decision making during treatment. Further research is required to refine and improve their capabilities, ensuring that they complement rather than replace the expertise of healthcare professionals.

## AUTHOR CONTRIBUTIONS

LR contributed to conceptualization, methodology, software, investigation, data curation, writing the original draft, writing—review & editing, and visualization. GL contributed to methodology, software, data curation, writing the original draft, and visualization. NM contributed to methodology, data curation, and writing—review & editing. DG contributed to conceptualization, methodology, writing—review & editing, and supervision. IL contributed to conceptualization, methodology, investigation, data curation, writing the original draft, writing—review & editing, visualization, supervision, and project administration.

## CONFLICT OF INTEREST STATEMENT

The authors have no conflicts of interest.

## Data Availability

Data sharing is not applicable to this article as no new data were created or analyzed in this study.
